# The Influence of Antibiotic-Loaded Bone Cement Spacer Type on Outcomes of the First Stage of a Revision Total Hip Arthroplasty

**DOI:** 10.3390/antibiotics14101034

**Published:** 2025-10-16

**Authors:** Gennaro DelliCarpini, Farouk Khury, Itay Ashkenazi, Katherine Shehadeh, Ran Schwarzkopf, Joshua C. Rozell, Nimrod Snir

**Affiliations:** 1 Department of Orthopedic Surgery, NYU Langone–Long Island, Mineola, NY 11501, USA; 2 Department of Orthopedic Surgery, NYU Langone Health, New York, NY 10003, USA; 3 Division of Orthopedic Surgery, Rambam Health Care Campus, The Ruth and Bruce Rappaport Faculty of Medicine, Haifa 31096, Israel; 4 Division of Orthopedic Surgery, Tel-Aviv Medical Center, Tel-Aviv 6423906, Israel

**Keywords:** total hip arthroplasty, two-stage revision, antibiotic spacer, mechanical complications

## Abstract

**Background:** There is no consensus on the optimal type of antibiotic-loaded bone cement (ALBC) spacer for ue in the first stage of revision total hip arthroplasty (THA) as a method for treating/managing periprosthetic joint infection (PJI) following THA. The purpose of this study was to determine the influence of ALBC spacer type on a collection of outcome metrics for revision THA (rHA). We hypothesized that infectious complications would be comparable across spacer types, while mechanical complications would be more common with the use of hemiarthroplasty ALBC spacer designs in rHA. **Methods:** This was a multicenter retrospective review of 144 patients who underwent a planned two-stage revision for THA for PJI between 2011 and 2022. Patients were stratified into three groups: pre-molded articulating hemiarthroplasty ALBC (PHA) spacers, custom articulating hemiarthroplasty ALBC (CHA) spacers, and custom ALBC THA (CTHA) spacers. The types and incidence of complications that arose during the time that the spacer was in situ were obtained. In total, 29 (20.1%) PHA, 11 (7.6%) CHA, and 104 (72.2%) CTHA patients were included. **Results:** CHA patients were significantly younger (mean age: 54.1 years) than PHA (mean age: 63.9) and CTHA (mean age: 63.9) patients (*p* = 0.011). The proportion of patients discharged home was significantly higher in the CHA group (81.8%) compared to that in both the CTHA group (64.4%) and the PHA group (34.5%) (*p* = 0.02). The re-revision rate for hip PJI was higher for CHA (18.2%), followed by PHA (13.8%) and CTHA (5.8%), while hemiarthroplasty spacers had the highest dislocation rate (13.8% in PHA and 18.2% in CHA vs. 3.8% in CTHA, *p* = 0.055). The vast majority of the outcome metrics did not differ among the three spacer groups, with examples being the incidence of dislocations, the incidence of all types of unplanned procedures for treating/managing re-infection, the time to the second stage in the rHA, and the proportion of spacers that were not removed until the second stage in the rHA. **Conclusions:** For the vast majority of the outcome metrics determined, the difference among the three study groups was not significant. Thus, the present results suggest that antibiotic-loaded cement spacer type does not influence outcomes when a spacer is used in the first stage of a two-stage protocol for the revision of a THA.

## 1. Introduction

Prosthetic joint infection is a feared complication following total hip arthroplasty (THA), conferring substantial patient morbidity [1,2,3,4]. Approximately 1–2% of all THA procedures involve the development of a periprosthetic joint infection (PJI) postoperatively [5]. PJI has historically portended longer hospital lengths of stay (LOSs), higher overall costs, and higher rates of repeat revision compared to those for non-infectious revision indications [4].

A two-stage exchange protocol with interim antibiotic spacer implantation remains the gold standard for the treatment of a chronic PJI [6]. During the first stage, the infected prosthesis is exchanged for a functional antibiotic-loaded bone cement spacer (ALBC) and provides high concentrations of local antibiotics [7]. This is typically followed by a second-stage procedure to remove the antibiotic spacer and replace it with a definitive, long-term prosthesis [8]. Although this is a well-accepted mode of treatment, there remains controversy regarding the spacer type used in the first stage [9].

ALBC spacers are classified as static or articulating, with a wide range of designs available. Static spacers do not allow for functional motion of the hip [10] but, as a result of a greater surface area, allow for a higher concentration of antibiotics and can be useful in the treatment of patients with severe bone loss [7]. Articulating spacers more closely recreate native hip anatomy and biomechanics and therefore maintain soft tissue tension, provide improved functional status while in situ, and improve the ease of the second-stage surgery [7]. Concerns with articulating spacers include increased complexity at the first stage, increased cost, the risk of spacer dislocation, and the potential for subsidence or fracture with weight bearing. Articulating spacers are designed in the form of either a hemiarthroplasty (HA), wherein the cement head directly contacts the bare acetabulum, or a custom THA (CTHA), wherein a metal or ceramic head articulates with a cemented ultra-high-molecular-weight polyethylene (UHMWPE) liner in the acetabulum. An articulating hemiarthroplasty spacer may be produced directly by a manufacturer (pre-molded) (PHA) or made custom by the surgeon intraoperatively (CHA) [9,11]. Prior evidence comparing different articulating spacer designs for hip PJI indicates that while infection eradication rates remain broadly comparable, superior mechanical and functional benefits are associated with more constrained or component-based designs. Specifically, real-component constructs have been shown to lead to a significantly smaller leg length discrepancy, lower dislocation rate, and higher likelihood of home discharge and trend toward a decreased length of stay compared to all-cement or hemiarthroplasty ALBC designs [6,12,13].

While the use of ALBC spacers for the treatment/management of PJI has been the subject of many studies [14], the relationship between spacer design, on the one hand, and infection profile and mechanical complications following first-stage revision, on the other hand, has not been adequately explored. Examples of infection complications are those that affect the skin/soft tissue and sepsis, whereas mechanical complications include dislocations and periprosthetic fractures. The purpose of the present study was to determine the influence of spacer type on the incidence of each of these complications, as well as other outcome metrics, such as the number of unplanned reoperations and unplanned revisions following first-stage revision of THA, in patients treated for PJI with planned two-stage revision, and spacer placement during the time the spacer was in situ. We hypothesized that while the influence of spacer type on the incidence of infectious complications would be marginal, it would be marked in cases of the incidence of mechanical complications.

## 2. Material and Methods

### 2.1. Study Design

Institutional review board approval was obtained prior to initiating the study. This was a multicenter, retrospective review of patients with a history of prior THA undergoing a planned two-stage revision THA (rTHA) for PJI between 1 July 2011 and 31 August 2022. Patients above 18 years of age with a diagnosis of PJI defined according to criteria previously published by the Musculoskeletal Infection Society (MSIS) who underwent at least the first stage of a planned two-stage rTHA with an articulating, antibiotic spacer were eligible for inclusion [15]. All surgical procedures were complete by high-volume orthopedic surgeons with subspecialty training in adult reconstruction.

**Figure 1:**
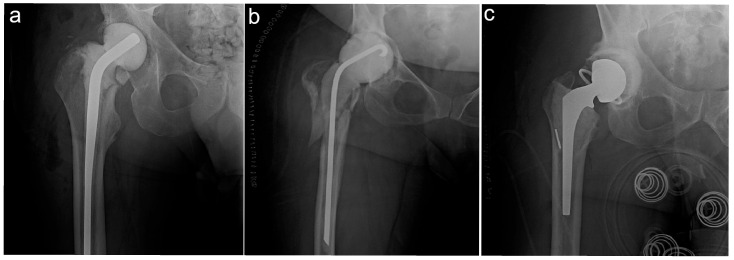
The different spacer types investigated in this study. (**a**) PHA—pre-molded hemiarthroplasty spacer; (**b**) CHA—custom hemiarthroplasty spacer; (**c**) CTHA—custom total hip arthroplasty spacer.

Patients undergoing revision for non-infectious indications, single-stage revisions, acetabular head and/or liner exchange or revisions with stem retention, debridement antibiotic and implant retention (DAIR) procedures, or non-articulating spacer/girdlestone procedures were not eligible. In total, 2039 revision procedures from our institutional databases were reviewed. Seven fellowship-trained arthroplasty surgeons completed the revision surgeries. After excluding 1567 aseptic revisions, 250 single-stage revisions, 47 acetabular liner and/or head exchanges with stem retention, 12 isolated DAIR procedures, and 19 non-articulating spacers/girdlestones, 144 planned two-stage rTHAs remained for final analysis. Spacer characteristics were extracted from the electronic medical record (EMR) and used to stratify patients into three groups: pre-molded hemiarthroplasty (PHA) spacers, custom hemiarthroplasty (CHA) spacers, and custom total hip arthroplasty (CTHA) spacers. Spacer type was selected based on the surgeon’s preference. Pre-molded spacers were manufacturer-prepared and fully formed, while custom spacers were fashioned by the surgeon intraoperatively. Pre-molded spacer types included the InterSpace (Exactech Inc., Gainesville, FL, USA) antibiotic spacer. For all CTHA spacers, a cemented acetabular component was placed, which was either constrained or unconstrained. Custom cemented stem types included Omnifit (Stryker, Kalamazoo, MI, USA), Secur-Fit (Stryker, Kalamazoo, MI, USA), and PROSTALAC (Depuy, Warsaw, IN, USA). The cement used for implantation was mixed with 2 g of vancomycin and 3.6 g of tobramycin per bag of cement. Figure 1 demonstrates the types of spacers used.

### 2.2. Baseline Demographics and Perioperative Characteristics

Patient baseline characteristics, such as age at revision procedure, body mass index (BMI), sex, self-identified race, smoking status, American Society of Anesthesiologists (ASA) class, and Charleson Comorbidity Index (CCI), were retrospectively extracted from EMRs. Perioperative characteristics at the time of the first stage, such as Paprosky classification of femoral bone loss, spacer femoral head diameters (mm), extended trochanteric osteotomy (ETO) utilization, operative time (min), hospital length of stay (LOS) (days), and discharge disposition, were also extracted from the EMRs.

### 2.3. Assessment of In Situ Spacer Complications

The study evaluation period began at the time of first-stage spacer implantation and extended through the duration for which the spacer remained in situ. Patient EMRs were accessed for information about complications throughout the duration the spacer remained in situ, with follow-up periods ending at the time the spacer was successfully explanted during the planned second stage or at the time of the latest clinical follow-up if spacer removal was deferred and the spacer was retained, at which point patients were censored from analyses. At final follow-up, the data obtained from the EMR included radiographic spacer subsidence; incidence of periprosthetic fracture; number of unplanned procedures for treating re-infection, including irrigation and debridement; and incidence of spacer exchange (other than for the planned second-stage revision). Final spacer outcomes were categorized as successful planned second-stage removal, in situ at latest follow-up, or unplanned spacer revision and compared across groups.

### 2.4. Data Analyses

Categorical variables were compared using Chi-squared analyses or Fisher’s exact tests with Freeman–Halton extensions, and continuous variables were compared using analyses of variance. Categorical variables were presented as a count (percentage), and continuous variables were presented as either the median and range (for head size) or the mean and range. The proportion of missing data for all variables was low and did not exceed the threshold [16,17] at which multiple imputation is generally recommended (approximately 50%). Consequently, each variable satisfied the criterion for reliable inclusion without the need for multiple imputation. Analyses were conducted using complete case data for each variable to ensure robustness and reduce potential bias. Homogeneity of variances for continuous variables analyzed with ANOVA was assessed using Levene’s test. All tests returned *p*-values > 0.05, confirming that the assumption of equal variances was met. Significance was set at *p* < 0.05. All data analyses were performed using IBM Statistical Package for the Social Sciences version 28 software (SPSS, Inc., Chicago, IL, USA).

### 2.5. Patient Characteristics

Of the total 144 patients included, there were 29 (20.1%) in the PHA group, 11 (7.6%) in the CHA group, and 104 (72.2%) in the CTHA group. Ten (9.6%) of the CTHA patients had a constrained acetabular component. The CHA group was younger (*p =* 0.011) and had a higher proportion of women (*p* = 0.047) compared to the PHA and CTHA groups. BMI, ASA class, and CCI were comparable across groups. Full demographic data are shown in Table 1.

## 3. Results

### 3.1. Perioperative Characteristics

Complete perioperative data are shown in Table 2. Paprosky class (*p* = 0.627) and ETO utilization (*p* = 0.483) were comparable across groups. Smaller head sizes were used in the CTHA group (32 mm [22 to 56]) compared to those in the CHA (50 mm [40 to 60]) and PHA groups (52 mm [46 to 60]) (*p* < 0.001). Surgical time was longest in the CHA (217.5 min [134 to 309]) group, followed by the CTHA (205.9 min [55 to 441]) and PHA (187.7 min [84 to 342]) groups, though these differences were not significant (*p* = 0.294). LOS was longest in the CHA (14.6 days [1.8 to 31]) group compared to that in the CTHA (6.2 days [1.8 to 35]) and PHA (6.4 days [2 to 16]) groups (*p* < 0.001). Discharge home was most common in the CHA group, followed by the CTHA and PHA groups (*p* = 0.02).

### 3.2. Evaluation of In Situ Spacer Complications

Data on complications while the spacer remained in situ are presented in Table 3. Considering all spacer dislocations, the rates of dislocation were highest in the CHA (*n* = 2, (18.2%)) group, followed by the PHA (*n* = 4, (13.8%)) and CTHA (*n* = 4, (3.8%)) groups, though significance was not achieved (*p* = 0.055). Among dislocations, return to the operating room was required in one of four PHA cases, one of two CHA cases, and in all four CTHA cases. All four CTHA cases necessitated return to the OR due to dissociation of the cemented liner/cup from the acetabulum. No periprosthetic fractures were observed in the CHA group, while 4.8% of CTHA patients and 10.3% of PHA patients sustained a periprosthetic fracture (*p* = 0.363). We did not note any cases of spacer fracture. Rates of unplanned procedures for infection were highest, in absolute numbers, among the CTHA group, though these differences did not reach significance (Table 3). Readmission for skin and soft tissue complications (*p* = 0.468), sepsis (*p* = 0.824), and other non-orthopedic indications (*p* = 0.600) was comparable across groups. Considering the final spacer outcomes, unplanned spacer revision rates were highest among CHA (*n* = 2, (18.2%)) patients, followed by PHA (*n* = 3, (10.3%)) and CTHA (*n* = 9, (8.7%)) patients (*p* = 0.593). Spacer preservation in situ at final follow-up was most commonly observed in the CTHA group (*n* = 17, (16.3%)), followed by the CHA (*n* = 1, (9.1%)) and PHA (*n* = 2, (6.9%)) groups (*p =* 0.382). In cases of successful second-stage spacer removal, the mean time for which a spacer was in situ was 5.5 months (range: 0.7–33.6 months). The time to second-stage surgery was comparable across groups (PHA: 10 ± 11.2 months, CHA: 13.2 ± 14.1 months, CTHA: 12.6 ± 12.8 months, *p* = 0.605).

## 4. Discussion

This multicenter study compared the outcomes between three types of articulating spacers used during the treatment of PJI after THA in 144 patients. The main findings were (1) a trend toward a higher incidence of dislocations observed for HA spacers compared to that in the custom THA group; (2) a trend toward increased unplanned reoperations for infection in the CHA group, followed by the PHA and CTHA groups; and (3) no significant difference in time to a successful second stage or rates of successful second-stage revision across groups.

PJI remains a serious complication following THA [2,4], often requiring two-stage exchange with an interim antibiotic spacer [7,18]. While articulating spacers offer functional and biomechanical advantages over static designs, their use introduces potential mechanical risks, especially in HA configurations [10]. Despite their widespread use, limited data exist comparing infection and mechanical outcomes among various forms of articulating spacers. To the best of our knowledge, this is the largest study conducted across multiple centers to evaluate and compare outcomes between different types of articulating spacers.

Although articulating spacers support ambulation, preserve soft tissue tension, and minimize contracture formation, they are also prone to dislocation, with reported rates as high as 42% [19,20]. While constrained acetabular liners have been proposed in cases of high dislocation risk, a recent study by Sarfraz et al. [12] reported no difference in dislocation rates based on the level of acetabular liner constraint. Giving these challenges, optimizing spacer design is critical in improving stability. CTHA implants may offer a closer replication of an uninfected THA, as they allow for modularity in head size, offset, and selection of the polyethylene cup. The surgeon can control the position of the cemented polyethylene acetabular liner and the femoral stem. In accordance with the changes in modularity and placement that THA components afford, the present study demonstrated a trend toward lower dislocation rates in the CTHA cohort (3.8%) compared to those in PHA (13.8%) and CHA (18.2%) patients (Table 3). This may be attributable to the ability to recreate normal biomechanics with accurate placement of the cup and soft tissue tensioning [21]. However, of note, head size and stem modularity was not controlled, which can impact the rates of dislocation when comparing spacer types. Although the CTHA group trended toward the lowest percentage of dislocation, when dislocation occurred, it required a return to the OR due to dissociation of the cemented liner/cup from the acetabulum, which may present a limitation to the use of CTHA. Despite this, one might argue that the use of constrained acetabular liners contributed to the reduced dislocation risk observed in CTHA. However, it is worth noting that only 9.6% of the CTHA cohort received a constrained acetabular liner. One possible explanation for the higher dislocation rates observed with PHA and CHA spacers is their mechanical similarity to non-cemented monoblock HA, in which rotational instability of the stem within the proximal femur has been implicated as a dislocation mechanism [22]. Although one previous study [23] hypothesized that proximal cementation of the femoral spacer might reduce this risk, multiple studies [24,25] have found no significant difference in dislocation rates between proximally cemented and uncemented designs. Furthermore, the first-stage debridement often results in proximal femoral bone loss, potentially compromising implant fixation and contributing to reduced spacer stability in HA constructs. Ultimately, dislocation is likely multifactorial [19,26], and future investigations should consider additional contributors such as spacer femoral neck–shaft angle [27], the degree of offset restoration [28], the level of patient ambulation [29], spacer head size [30], and underlying diagnosis leading to primary THA, as well as muscular insufficiency, limb length discrepancy, and the extent of bone loss [24].

While direct comparative data are scarce, it is plausible that surgeons may favor PHA or CHA spacers in scenarios such as challenging femoral anatomy, severe acetabular bone loss that precludes polyethylene liner use, patient frailty where shorter operative times are preferred, or when resource constraints limit access to more complex constructs [31]. In contrast, CTHA spacers offer several potential advantages, including the ability to utilize semi-constrained or constrained liners, the selection of femoral stems that surgeons are already familiar with, and protection of the acetabulum from further bone erosion that can occur with hemiarthroplasty designs [32]. Additionally, some surgeons prefer to avoid introducing an additional UHMWPE articulation in patients at high re-infection risk because polymeric surfaces (including UHMWPE) may be comparatively more susceptible to bacterial adhesion and biofilm formation [33,34]. In our series, no clear chronological trend was observed in spacer selection, with PHA, CHA, and CTHA spacers used throughout the study period; this stable utilization over time likely reflects surgeon preference, patient-specific considerations, and institutional/resource factors rather than an evolution toward exclusive CTHA use.

The current rates of successful infection eradication in chronic PJI are promising, with over 80% of patients remaining infection-free after two-stage surgery in the previous literature [21,35,36,37]. We found that the rates of unplanned procedures for infection were similar between groups, including the CHA group (18.2%), followed by PHA (13.8%) and CTHA (6.5%) patients (*p* = 0.181). Custom-made implants have the greatest degree of variability in the amount of antibiotic added during implantation, while pre-molded spacers have a standardized quantity of antibiotic in the spacer construct [7]. Furthermore, antibiotic elution has been shown in previous studies to peak in the first 24 h after implantation, which demonstrates the limited sustained effect and impact of spacer-mediated local antibiotic delivery [14]. Regardless of the type of implant used, it is not uncommon for patients to return to the operating room due to infection-related complications in between stages [38]. Biomaterials may serve as a substrate for bacterial adhesion and biofilm formation. Utilizing THA spacers introduces an additional surface for biofilm formation due to the acetabular component [36]. These components may harbor bacteria and reduce the overall efficacy of articulating spacer treatment. As seen in a study by Karbysheva et al. [39], bacterial contamination in retrieved polyethylene components is common after two-stage exchange. However, in our study, CTHA spacer designs trended toward the lowest rates of reoperation for infection, and the use of antibiotic cement in the acetabulum may be important to mitigating this additional risk. This has been observed in a previous study [6].

There was no difference in the overall time to reimplantation in our study between spacer types (1.7 to 33.6 months, *p* = 0.605, Table 3). Though over 70% of patients in all groups underwent a second-stage revision, the CTHA group (16.3%) had the largest proportion of patients with spacers remaining in situ at the latest follow-up, compared to CHA (9.1%) and PHA (6.9%) patients (Table 3). Previous studies [11] have shown that subsets of patients have refused further surgery due to satisfaction with their spacer. Petis et al. [40] retrospectively reviewed patients undergoing two-stage revision for PJI and reported 17 cases with a retained spacer and 47% of patients retaining the spacer due to good overall function. However, long-term studies on spacer retention have yet to be performed.

Although the CHA patients were significantly younger and had a higher chance of being discharged home after the study (Table 1 and Table 2), they had a significantly longer LOS (14.6 days) (*p* < 0.001, Table 2). However, the discharge disposition of the CHA group may be influenced by their significantly lower age, which may represent an overall healthy cohort more likely to be discharged home. This data may suggest that certain spacers may hinder patient progression both out of the hospital and home, which can increase the overall cost of treatment. Of the three spacer types investigated, CTHA patients had the shortest LOS (6.2 days). Furthermore, the majority (64.4%) of CTHA patients were discharged home postoperatively. These findings may indicate better comfort and ability to perform activities of daily living due to improved function and less pain with the use of a custom THA spacer. This has been noted in prior studies [11] on articulating spacers with the use of an acetabular component. In terms of the operative time difference, despite the increased modularity and complexity of custom THA spacer placement, there were no significant differences in operative time between the spacer groups (205.9 min in CTHA vs. 217.5 min in CHA vs. 187.7 min in PHA, *p* = 0.294, Table 2). This is in contrast to Kugelman et al. [6], who reported longer operative times for CTHA spacers when compared to hemiarthroplasty spacers (217 vs. 181 min, *p* = 0.031). On the other hand, a study by Nahhas et al. [9] compared the operative times between various articulating and static spacer types, finding no difference. Our findings suggest that the additional technical steps of custom spacer placement may not contribute to the overall operative time. This may have been a consideration previously when selecting an implant during first-stage revision.

This study has several limitations. First, this study was retrospective, which means that selection bias and bias due to loss to follow-up may have been introduced. Loss to follow-up was seen in 20 patients who had their spacer in situ at latest follow-up, and it is unknown whether they underwent a second-stage revision elsewhere. Furthermore, we did not report the reason for some patients not returning for second-stage surgery, which is difficult to ascertain given the retrospective design. The reasons for retention are likely multifactorial, and the absence of a documented clear medical or surgical contraindication in the electronic medical record does not preclude its existence. Second, given the retrospective design, we could not control for the baseline characteristics of each cohort, including age (younger patients were present in the CHA cohort with an average age of 54.1), the percentage of male patients (significantly more males were present in the CTHA group (68.3%), and the race distribution of the patients. We also did not separate factors in the CCI, which was used to compare cohorts, as some individual conditions in the scoring system (such as diabetes) may contribute more to a worse outcome. Additionally, although the dataset was missing less than 50% of our data, there may have continued to be bias with interpretation, particularly if the missing data were not random secondary to the retrospective design. This may be improved upon with a multivariate analysis to elucidate further which factors contribute most to worse outcomes. Third, due to the retrospective nature of this study, we were unable to control the number of patients in each group. The CHA and PHA groups were smaller than the CTHA group; therefore, the conclusions from the smaller subgroup analysis may be subjected to bias. Fourth, data from multiple surgeons were used without standardization of features such as the chemistry and dosage of antibiotic-loaded bone cements in the spacer; the implant designs used, including head size; or postoperative rehabilitation regimens with the spacer in situ. This may be a confounding factor given that certain spacers may be used for a patient with a specific anatomical deformity and the spacer used was not controlled for. Fifth, there is inherent surgeon bias in the type of spacer used, the head size of the spacer, and the use of a constrained liner, influenced by factors such as host status, body habitus, bone loss, and overall surgeon preference toward a particular spacer type. Sixth, we did not report acetabular bone loss, which may also influence spacer selection, mechanical complications, and second-stage complexity. Seventh, our cohort demonstrated longer interim spacer durations than the conventional 6–12 weeks (5.5 months). This finding, which may reflect both institutional protocols (infection control evaluation, repeat aspiration, patient preference, and operative scheduling) and variability, has also been reported in the literature [41,42,43,44] without a clear impact on outcomes. Eighth, the cost of each of the spacer types was not obtained, which is a significant factor in treatment decisions, particularly since the median cost reported in the literature of CTHA was estimated to be up to USD 15,246, significantly more expensive than PHA (median: USD 1529) and CHA (median: USD 916) [45]. Despite the trend toward lower dislocation rates and rates of return to the operating room seen in the CTHA cohort, hospitals and surgeons may not choose to utilize this type of spacer option given the reported larger associated implant costs, without any significant improved benefit. The spacer selection should be individualized for each patient to optimize clinical outcomes, facilitate subsequent surgery, and minimize in situ complications. Ninth, patient- and organism-specific risk factors for complications, such as surgical characteristics and bacterial organism type, were not investigated. Such factors are crucial in understanding the complex etiology of complications in PJI treatment, and their omission limits the depth of our analysis. Tenth, due to the nature of the intervention investigated, this study may have been underpowered to detect differences in outcomes across spacer groups. Future large-scale, prospective studies are warranted to further evaluate long-term outcomes, with functional outcomes including pain, mobility, quality of life, and cost effectiveness, across spacer types for the treatment of PJI.

## 5. Conclusions

For the vast majority of the outcome metrics determined, the difference among the three study groups was not significant. Thus, the present results suggest that ALBC spacer type does not influence outcomes when a spacer is used in the first stage of a two-stage protocol for revision THA to treat/manage a PJI.

## Figures and Tables

**Table 1:** Baseline demographics stratified by spacer type.

	Revision Type			*p*-Value
Parameter	PHA (*n =* 29)	CHA (*n* = 11)	CTHA (*n* = 104)	
Age [range], (years)	63.9 [39 to 84]	54.1 [34 to 76]	63.9 [44 to 87]	0.011 *
BMI, (kg/m^2^)	30.8 (20.8 to 56.84)	34.5 (18.3 to 51)	30.9 (17.17 to 48.13)	0.308
Sex, n (%)				
Female	14 (48.3%)	7 (63.6%)	33 (31.7%)	0.047 *
Male	15 (51.7%)	4 (36.4%)	71 (68.3%)	
Race, n (%)				
White	18 (62.1%)	8 (72.7%)	79 (76%)	0.033 *
Black	8 (27.6%)	1 (9.1%)	8 (7.7%)	
Asian	1 (3.4%)	0 (0%)	0 (0%)	
Other	2 (6.9%)	2 (18.2%)	17 (16.3%)	
Smoking status, n (%)				
Never	18 (62.1%)	7 (63.6%)	44 (45.4%)	0.446
Former	7 (24.1%)	3 (27.3%)	39 (40.2%)	
Current	4 (13.8%)	1 (9.1%)	14 (14.4%)	
ASA class, n (%)				
I	3 (13.6%)	1 (10%)	2 (2%)	0.093
II	5 (22.7%)	6 (60%)	46 (46.9%)	
III	14 (63.6%)	3 (30%)	49 (50%)	
IV	0 (0%)	0 (0%)	1 (1%)	
Mean CCI [range]	2.9 [0 to 9]	3.1 [0 to 7]	3.1 [0 to 9]	0.882

n, number; PHA, pre-molded hemiarthroplasty; CHA, custom hemiarthroplasty; CTHA, custom total hip arthroplasty; BMI, body mass index; ASA, American Society for Anesthesiologists; CCI, Charleson Comorbidity Index; *, significance at *p* < 0.05.

**Table 2:** Perioperative characteristics stratified by spacer type.

	Revision Type			*p*-Value
Parameter	PHA (*n* = 29)	CHA (*n* = 11)	CTHA (*n* = 104)	
Paprosky class, n (%)				
I	10 (34.5%)	4 (36.4%)	41 (40.2%)	0.627
II	10 (34.5%)	3 (27.3%)	28 (27.5%)	
IIIa	6 (20.7%)	2 (18.2%)	27 (26.5%)	
IIIb	3 (10.3%)	1 (9.1%)	4 (3.9%)	
IV	0 (0%)	1 (9.1%)	2 (2%)	
Median head diameter [range], (mm)	52 (46 to 60)	50 (40 to 60)	32 (22 to 56)	<0.001 *
ETO, n (%)	12 (41.4%)	4 (36.4%)	31 (29.8%)	0.483
Mean surgery duration [range], (min)	187.7 (84 to 342)	217.5 (134 to 309)	205.9 (55 to 441)	0.294
Mean length of stay [range], (days)	6.4 (2 to 16)	14.6 (1.8 to 31)	6.2 (1.8 to 35)	<0.001 *
Discharge disposition, n (%)				
Home	10 (34.5%)	9 (81.8%)	67 (64.4%)	0.02 *
Acute rehab facility	3 (10.3%)	1 (9.1%)	7 (6.7%)	
Skilled nursing facility	16 (55.2%)	1 (9.1%)	30 (28.8%)	

n, number; PHA, pre-molded hemiarthroplasty; CHA, custom hemiarthroplasty; CTHA, custom total hip arthroplasty. ETO, extended trochanteric osteotomy. *, significance at *p* < 0.05.

**Table 3:** Complications with spacer in place stratified by spacer type.

	Revision Type			*p*-Value
Complications While Spacer In Situ	PHA (*n* = 29)	CHA (*n* = 11)	CTHA (*n* = 104)	
Subsidence, n (%)	6 (20.7%)	3 (30%)	19 (19.4%)	0.730
Dislocation, n (%)				
All	4 (13.8%)	2 (18.2%)	4 (3.8%)	0.055
Return to OR required	1 (3.4%)	1 (9.1%)	4 (3.8%)	0.693
Non-operatively treated	3 (10.3%)	1 (9.1%)	0 (0%)	0.005
Periprosthetic fracture, n (%)				
All	3 (10.3%)	0 (0%)	5 (4.8%)	0.363
Return to OR required	1 (3.4%)	0 (0%)	2 (1.9%)	0.774
Non-operatively treated	2 (6.9%)	0 (0%)	3 (2.9%)	0.468
Unplanned procedure for re-infection, n (%)				
All	4 (13.8%)	2 (18.2%)	6 (5.8%)	0.181
Requiring I&D	4 (13.8%)	1 (9.1%)	5 (4.8%)	0.232
Requiring spacer exchange	1 (3.4%)	1 (9.1%)	3 (2.9%)	0.565
Other complications requiring readmission, n (%)				
Skin/soft tissue	2 (6.9%)	0 (0%)	3 (2.9%)	0.468
Sepsis	0 (0%)	0 (0%)	1 (1%)	0.824
Other non-orthopedic	2 (6.9%)	1 (9.1%)	14 (13.5%)	0.600
Spacer outcome, n (%)				
Removed in second stage	24 (82.8%)	8 (72.7%)	78 (75%)	0.655
In situ at latest follow-up	2 (6.9%)	1 (9.1%)	17 (16.3%)	0.382
Unplanned spacer revision	3 (10.3%)	2 (18.2%)	9 (8.7%)	0.593
Time to second Stage ± SD [range], (months)	10 ± 11.2 [2.3 to 33.6]	13.2 ± 14.1 [1.7 to 33.6]	12.6 ± 12.8 [1.7 to 33.6]	0.605

n, number; PHA, pre-molded hemiarthroplasty; CHA, custom hemiarthroplasty; CTHA, custom total hip arthroplasty; I&D, irrigation and debridement.

## Data Availability

The data that support the findings of this study are not publicly available.
